# 

**Published:** 1894-05-12

**Authors:** 


					?k|r *| ? ?* THE KING OF NATURAL TABLE WATERS. ((T -| ? *?
uo^annia tH| doJpinia
No. 398. Vol. XYI.
SATURDAY
May 12th, 1894.
WITH EXTRA nuksinu sumtrntm. Vri?,f.."
[Registered at the General Post Office as a Newspaper for Monthly Parts, 6d.; post free, 8d.
transmission in the United Kingdom and Abroad.] Ditto per Annnm, 8s., post free.

				

